# Decomposition Kinetics and Lifetime Estimation of Thermoplastic Composite Materials Reinforced with rCFRP

**DOI:** 10.3390/ma17092054

**Published:** 2024-04-27

**Authors:** Juana Abenojar, Gladis Miriam Aparicio, José Antonio Butenegro, Mohsen Bahrami, Miguel Angel Martínez

**Affiliations:** 1 Materials Science and Engineering Department, Universidad Carlos III of Madrid, 28911 Leganés, Spain; jbuteneg@ing.uc3m.es (J.A.B.); mbahrami@ing.uc3m.es (M.B.); mamc@ing.uc3m.es (M.A.M.); 2 Mechanical Engineering Department, Universidad Pontificia Comillas, 28015 Madrid, Spain; 3 Basic Sciences Faculty, Autónoma of Occidente University, Calle 25, Cali 764007, Colombia; gmaparicio@uao.edu.co

**Keywords:** lifetime, decomposition, thermoplastic resin, polyamide 11, polyamide 12, reuse of carbon fiber composites

## Abstract

Because of the high demand for carbon fiber reinforced polymer (CFRP) materials across all industries, the reuse and/or recycling of these materials (rCFRP) is necessary in order to meet the principles of the circular economy, including recycling and reuse. The objective of this study is to estimate the lifespan of thermoplastic matrix composite materials reinforced with waste materials (CFRP), which undergo only a mechanical cutting process. This estimation is carried out through the thermal decomposition of polymers, including polymer matrix composite materials, which is a complex process due to the numerous reactions involved. Some authors calculate these kinetic parameters using thermogravimetric analysis (TGA) as it is a quick method, and it allows the identification of gases released during decomposition, provided that the equipment is prepared for it. This study includes a comparison between polyamides 11 and 12, as well as between polyamide composite materials with carbon fiber (CF) and polyamides reinforced with CF/epoxy composite material. The latter is treated with plasma to improve adhesion with polyamides. The behavior of weight as a function of temperature was studied at speeds of 3, 6, 10, 13, 17, and 20 °C/min, finding stability of the polyamides up to a temperature of 400 °C, which was consistent with the analysis by mass spectroscopy, where gas evolution is evident after 400 °C. The estimation of the lifespan was carried out using two different methods including the Toop equation and the free kinetics model (MFK). The energy of the decomposition process was determined using the MFK model, which establishes the energy as a function of the degree of conversion. It is estimated that at 5% decomposition, mechanical properties are lost.

## 1. Introduction

Carbon fiber-reinforced polymer (CFRP) composite materials are highly regarded for their unique properties, combining a polymer matrix with carbon fiber reinforcement [1]. These materials offer high mechanical strength, low weight, stiffness, resistance to wear, corrosion, and heat, as well as a low coefficient of thermal expansion [2]. Such quality properties have led to a significant impact across various industries, particularly aerospace, automotive, construction, energy generation, and sports equipment production.

In aerospace, CFRPs are transforming aircraft construction [3], while in the automotive sector, they are enhancing chassis strength and reducing weight for electric vehicles [4,5]. Similarly, the construction industry is gradually embracing CFRPs for infrastructure projects [6,7]. Additionally, CFRPs hold promise in energy generation and sports equipment production [8,9]. Anticipated growth in adoption across various industries underscores the versatility and potential of CFRP materials.

Thermoset matrices, such as epoxy and polyester resins, are key components in CFRPs. They boast characteristics like low viscosity and hydrophilic properties, facilitating good wettability. Their chemical curing reaction, initiated by various active groups, is exothermic and results in robust crosslinking, with reaction kinetics dictating curing duration [10,11]. Other less common thermoset matrices include vinyl ester, phenolic, and polyimide resin.

Thermoplastic matrices, despite their lower thermal resistance and viscosity compared with thermosets, offer unique advantages. They can be melted and reshaped multiple times without losing properties, thus aiding in recycling. Additionally, they provide greater toughness, impact resistance, dimensional stability, and environmental resistance [12]. Composites with thermoplastic matrices often have shorter curing times, enhancing production efficiency for complex components. Carbon fiber-reinforced thermoplastic polymer (CFRTP) composites, a subset of CFRP, combine carbon fiber strength with thermoplastic flexibility. CFRTPs offer speedier manufacturing, recyclability, and superior impact resistance compared with traditional CFRPs. Thus, they find diverse applications in industries requiring strength, stiffness, durability, recyclability, and ease of fabrication [13,14,15].

This study employs polyamides 11 and 12 (PA11 and PA12) as thermoplastic matrices because of their well-documented mechanical properties and compatibility with rCFRP [16]. PA11, a bio-based polyamide, offers good thermal resistance, chemical resistance, and low environmental impact. PA12, a synthetic polyamide, excels in low-temperature strength, rigidity, and various resistance properties.

The pursuit of a circular economy and sustainable resource utilization has highlighted the critical role of recycling composite materials, particularly the reuse of carbon fiber composites, known for their desirable strengths and lightweight characteristics [17]. This necessity has pushed the focus on the decomposition kinetics and lifetime estimation of composite materials, specifically thermoplastic composites reinforced with recycled carbon fiber reinforced polymer (rCFRP). Understanding the thermal degradation behavior and predicting the service life of such composite materials are vital for ensuring their integrity and reliability in diverse industrial applications.

Thermogravimetry (TGA) is especially useful for understanding how composite materials decompose or degrade as a function of temperature, which can help optimize their performance and durability in various applications [18]. Additionally, this technique can be used to assess material purity, identify additives or impurities, and characterize the kinetics of the thermal decomposition of materials [19,20,21,22,23]. TGA analysis, which is a rapid method, can determine the thermal stability of many materials. For polymers, pyrolysis occurs during the process, revealing the gases released during decomposition [24]. However, this requires specialized equipment.

When the direct explanation of thermal properties and their variations are not immediately accessible, kinetic models step in to bridge the gap. Over the years, several methods and models have been developed to analyze the kinetics of decomposition and estimate the lifetime of composite materials. Based on the chemical reaction rate theory and principles of the Arrhenius law [25], they provide an empirical means of reviewing material properties as they change under the influence of temperature and heating rates.

The kinetics of composites, particularly those under thermal analysis, yield essential parameters that guide predictions about their behavior and reactions. Vyazovkin et al. [26] and Yu et al. [27] summarized methods like Ozawa, Friedman, Kissinger, and the modified Coats–Redfern for their applicability to Polymer Matrix Composites (PMCs), offering prescriptive advice for those traversing the complexities of these kinds of analyses. In this context, “multi-curve” methods such as the Friedman [28] and Ozawa [29] methods determine a set of kinetic model parameters (KMPs) across a range of heating rates. Meanwhile, “single-curve” methods like the modified Coats–Redfern [30] provide for the derivation of these kinetic parameters from TGA data at a unique heating rate. Selecting the appropriate model depends on the particular thermal behavior and kinetics of degradation of the composites that are being studied.

The decomposition kinetics of composite materials have been the subject of extensive research by the scientific community, offering a wide range of models and methods. For instance, the study by Zhang et al. [31] employed the Coats–Redfern and Flynn–Wall-Ozawa methods to study basalt fiber-reinforced polyethylene composites, unveiling enhanced thermal stability with basalt fibers. Enciso et al. [32] combined the Kissinger model and model-free kinetics (MFK) to assess the degradation behaviors of natural fibers and polyethylene composites, using the Toop equation for lifetime predictions. The MFK model, which Mettler applied to kinetic software, is based on the theories of Vyazovkin and Coats–Redfern [33,34]. Based on the degree of conversion, this model calculates the energy [35].

Similarly, Sihn et al. [36] emphasized the precision achievable by using a unified approach to kinetic modeling for polymer composites. Batista et al. [37] used the Flynn–Wall-Ozawa and Kissinger methods to determine the activation energy for poly(ether imide)/carbon fiber composites and estimated their lifetimes through the Arrhenius equation. In contrast, Liao et al. [38] incorporated multiple methods to predict the service life of stranded carbon fiber composite core conductors, setting the stage for the application of these composites in the demanding sphere of overhead transmission.

To the best of the authors’ knowledge, this study represents the first investigation of mechanically recycled carbon fiber (rCF) as a reinforcement material in combination with PA11 and PA12 thermoplastic matrices. This innovative approach offers a sustainable and potentially cost-effective alternative to virgin carbon fibers, promoting environmental benefits through material reuse (reduced landfill waste and CO_2_ emissions). These novel composites derived from discarded materials are investigated for lifetime estimation, focusing on their thermal degradation behavior.

In this regard, this study delves into the complex dynamics of decomposition kinetics and the estimation of lifetime in thermoplastic composite materials reinforced with rCFRP, addressing the imperative to extend the life cycle of such materials while maintaining their performance and sustainability. The investigation proceeds by interpreting TGA data through the prism of the Kissinger method, the MFK model, and the Toop equation, aiming to interpret the complicated kinetics of decomposition. Moreover, within the framework of this investigation, we consider the atmospheric plasma treatment’s effects on reinforcement, seeking insights into how surface treatments influence decomposition energy.

The exploration of these essential aspects within the context of composite materials not only contributes to advancing material science but also aligns with the broader objectives of environmental conservation and resource optimization.

## 2. Materials and Methods

### 2.1. Materials

Commercial pellets of PA11 and PA12 were provided by Arkema (Madrid, Spain). Bidirectional carbon fiber fabrics were utilized with an areal density of 600 g/m^2^ produced by Materiales Estructurales Ligeros S.L. (Barcelona, Spain). The chosen material for CFRP consisted of pultruded carbon fiber plates (Carbodur S 512, Sika S.A.U., Alcobendas-Madrid, Spain). This composite was composed of carbon fiber and epoxy resin, with a density of 1.60 g/cm^3^, a fiber volume fraction exceeding 68%, a tensile strength of 2900 MPa, a strain at failure of 1.80%, and a glass transition temperature above 100 °C [39].

The expired composite plates were mechanically cut to obtain sticks with a length of 40 mm to be used as reinforcement while maintaining the original thickness of 1.2 mm. Therefore, the reinforcement serves as an example of mechanically recycled and reused CFRP, referred to as CF + EP.

### 2.2. Plasma Treatment

The CF + EP surface was modified through low-pressure plasma (LPP) treatment to improve adhesion with the matrix (CF + EP LPP). LPP treatment, detailed in a previous study, utilizes a plasma chamber (Harrick, Ithaca, NY, USA) to generate reactive species that modify surface chemistry. This treatment was conducted at 300 m Torr, 30 W for 2 min to enhance adhesion, which is critical for optimal composite performance. To maintain the effectiveness of the treatment, the composite material fabrication immediately followed plasma treatment.

### 2.3. Wettability Measurement

After the plasma treatment, a DataPhysics OCA15 Plus goniometer (DataPhysics Instruments GmbH, Filderstadt, Germany) along with SCA20 software V1.0 (DataPhysics Instruments GmbH, Filderstadt, Germany) was used for contact angle measurements to assess the matrix wettability on carbon fibers or reinforcements. The tests involved using water, glycerol, and diiodomethane as liquids, with or without resin. Following the contact angle measurements, the Owens–Wendt–Rabel–Kaelble (OWRK) model [40,41] was employed to determine the surface energy and its components based on the surface tension components of test liquids.

### 2.4. Manufacturing of Composite Materials

PA pellets were filled inside a steel mold, which was then sandwiched between two aluminum sheets of 2 mm thickness and two steel plates of 3 mm thickness to produce bulk and sheets of polyamides with 4 mm and 1 mm thickness, respectively. The PA pellets were melted in a hot press with a maximum temperature and pressure of 200 °C and 45 kN for 60 min. Gradual pressure steps were utilized to obtain better densification and to eliminate trapped air between pellets. The same cycle was employed for composites, resulting in laminates of PA/CF/PA/CF/PA, for PA11_2CF and PA12_2CF.

CF + EP or CF + EP LPP composites were manufactured by placing the reinforcement on the polyamide sheet while maintaining the one-dimensionality of the fiber. Fifty grams of reinforcement were evenly distributed manually on top and bottom of the manufactured polyamide sheet, sandwiched between aluminum plates within a steel frame, and underwent another round of hot-pressing to produce the final composite sheet.

### 2.5. Thermogravimetric Analysis (TGA)

This analysis is based on measuring the mass variation in a sample when subjected to a ramp of temperatures under a controlled atmosphere; in this case, N_2_ gas was used. The technique was used to determine the degradation temperature from 30 to 600 °C at a heating rate of 10 °C/min. The study of reaction kinetics and the estimation of the service life of composite materials were also conducted using TGA. The TGA Q500 from TA Instruments (New Castle, DE, USA) was used for these tests, equipped with a built-in mass spectrometer MS Discovery (TA Instruments) to obtain the molecular weights of the compounds released during the test and the temperature at which they do so. For each material, six tests were conducted at different heating rates (3, 6, 10, 13, 17, and 20 °C/min).

#### Kinetic Models Utilized to Calculate Decomposition Energy and Lifespan in Service

Using STAR software V 12.10 from Mettler Toledo and based on the thermograms obtained for each rate, it is possible to calculate the decomposition energy (E_d_) as a function of the material’s degradation degree (α) using the kinetic model incorporated in the software, MFK. This result can then be compared with that obtained using the Kissinger model [42]. In the Kissinger model, the activation energy is calculated from the peak temperature of the decomposition (T_p_) curve derivative as a function of the heating rate, according to Equation (1):

(1) $$\ln\left(\frac{\beta }{T_{p}^{2}}\right)=LnC-\frac{E_{a}}{RT_{p}}$$

where β is the heating rate, T_p_ is the temperature (K), R is the gas constant (8.314 J/mol K), and E_a_ is the activation energy equivalent to decomposition energy (J/mol) to be calculated.

By applying the equation proposed by Toop [43], Equation (2), it becomes possible to calculate the service life of each material as a function of temperature [44].

(2) $$\ln t_{f}=\frac{E_{a}}{RT_{f}}+\ln\left(\frac{E_{a}}{\beta R}\cdot P(X_{f})\right)$$

where β is the heating rate, R is the gas constant, and E_a_ is the decomposition energy calculated for the degree of decomposition obtained through the Kissinger equation (Equation (1)) considered as the failure of the material, which, in this case, is 5%. T_f_ is the chosen service temperature for calculating the lifespan and P(X_f_) is a function given by the values E_a_/RT_c_ (where T_c_ is the temperature at the percentage of decomposition considered as failure, 5%) according to Toop’s theory. Finally, t_f_ represents the estimated time to failure of the material, i.e., its service life.

## 3. Results

### 3.1. Decomposition Kinetics

Predicting the service life of materials is crucial in the design and projection of new components, but it can be a challenging task. Thermogravimetric analysis, combined with the Toop equation (Equation (2)), offers a rapid and effective method for estimating the service life of polymeric materials.

**Figure 1:**
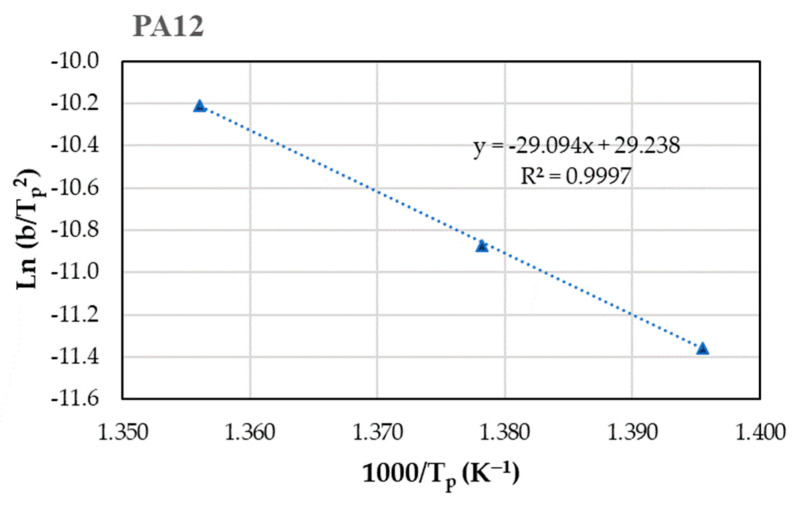
Obtaining E_d_ for PA12 from the Kissinger equation. Dashed line corresponds to the trend line, whose equation is presented in the figure itself.

TGA analysis was carried out on the polymeric matrices, PA11 and PA12, and on the composites of CF and CF reinforced with epoxy (CF+EP) at six different heating rates. From the values of the temperature at the peak of the decomposition curve derivative (Table 1) and the test rates, the energy of the process was calculated according to Equation (1) using the Kissinger model (Arrhenius-based equation) based on the slope of the line, as illustrated in Figure 1. The obtained values for each material are shown in Table 2.

**Figure 2:**
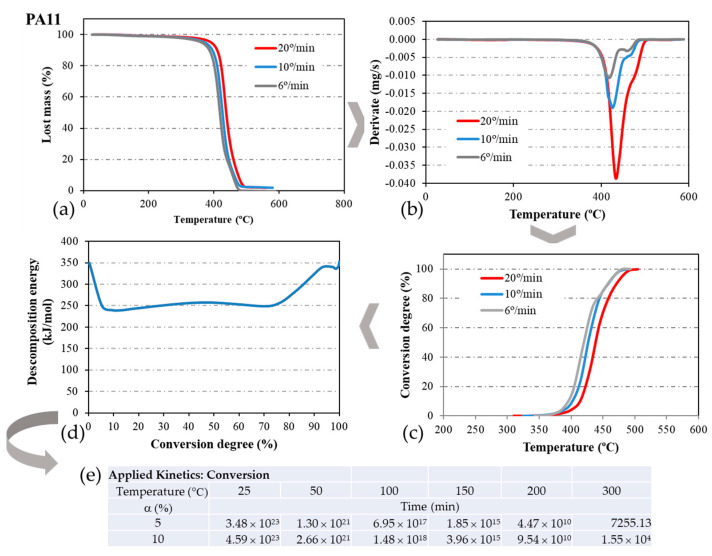
E_d_ calculation process of the PA11 decomposition process according to the MFK model. (**a**) TGA curves, (**b**) derivatives of TGA curves, (**c**) degree of decomposition, (**d**) decomposition energy, and (**e**) isothermal simulation.

Although the decomposition of the materials occurs in the temperature range of 400 °C < T < 500 °C (Figure 2a), Table 1 presents the peak of decomposition temperature (Figure 2b) as a result of the maximum change that experiences the variation in the weight of the sample (T_d_). The values in Table 1 represent the mean value of the four TGAs carried out for each material. The PA11 polymer presents a T_d_ value 28° lower than that of PA12, representing a 6% difference. For the composites with 2CF, the difference is 7% between PA11 and PA12. In the case of composites with recycled material, the difference increases to 37° for those with a PA12 matrix, representing an 8% difference. PA11 consists of only one monomer, aminoundecanoic acid, with the amine group at one end and the acid group at the other. It polymerizes with itself to form chains with 11 carbons between the two nitrogen atoms. On the other hand, PA12 originates from aminolauric acid, also having the amine group at one end and the acid group at the other. Polymerizing with itself results in polyamide containing 12 carbons between the two nitrogen atoms of the amine groups. Consequently, the PA12 chain is longer, which may lead to a higher T_d_ than PA11.

Based on the data presented in Table 2, it can be observed that for PA11 and its composites, the CF reinforcement does not cause an increase in E_d_. However, for the CF + EP composite, E_d_ decreases by approximately 42 kJ/mol (15%) and remains in the same range as PA11 when treated with LPP. In the case of PA12, when CF is added, E_d_ remains similar to the pure polyamide, but it increases by 20 kJ/mol for CF + EP (8%), while E_d_ remains similar to PA12 when LPP treatment is performed on CF + EP. This decrease in E_d_ may be attributed to the porosity that can form around the fibers because of a lack of adhesion, making the decomposition process easier.

It should be noted that with this model, a single E_d_ is assumed for the entire process with an n-order mechanism with n = 1, which is unlikely for this type of reaction. Furthermore, as empirical models, differences of this magnitude cannot be considered excessive.

To validate the hypotheses derived from the Kissinger model, we employed another kinetic model that calculates the energy of the process based on the degree of conversion. Known as “Model Free Kinetics” (MFK), this model is integrated into STARe software V12.10. For each material, the thermograms from the TGA analysis (Figure 2a) were derived (Figure 2b) to obtain the conversion curves (Figure 2c), and from these, the energy of the decomposition reaction as a function of the degree of conversion (Figure 2d), following the process outlined in Figure 2. All thermograms exhibited a weight loss at approximately 5%, occurring around 400 °C, signifying the onset of material degradation.

The program enables estimation of the material’s lifespan at one or multiple temperatures and various degrees of conversion, simulating an isothermal cycle at these temperatures, as illustrated in Figure 2e. This estimated time can be compared with that obtained using the Toop equation. It is important to note, however, that the estimation error is quite high, particularly at the onset and conclusion of the reaction, reaching approximately 10%.

**Figure 3:**
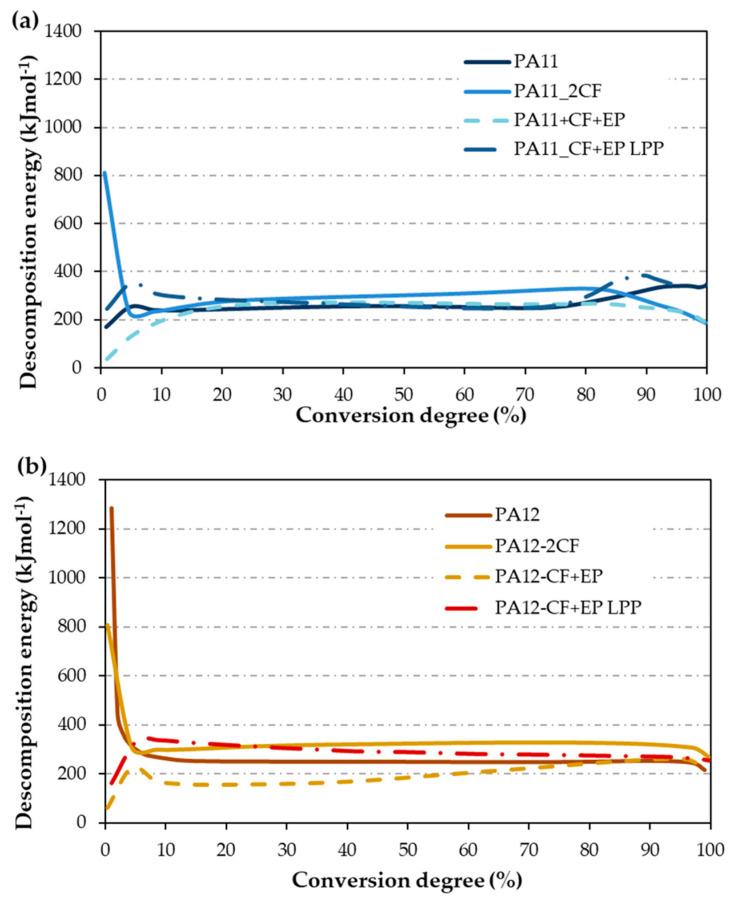
Decomposition energy as a function of the degree of decomposition of the composites (**a**) PA11 and (**b**) PA12.

The curves depicting the decomposition energy (E_d_) for PA11 composite materials in comparison to the PA11 matrix are illustrated in Figure 3a. The average E_d_ value aligns within the same range as that obtained using the Kissinger method in the central segment of the curve (50% decomposition). While the Kissinger method offers a quicker application and provides a reasonably close approximation of E_d_, the MFK model offers greater control, especially at the initial stages of the reaction, revealing more nuances in the decomposition process. Consequently, the decomposition reaction proves to be more intricate, deviating from a simple first-order reaction assumption made by the Kissinger method, necessitating a more in-depth investigation.

The energy required to initiate the degradation reaction varies based on several factors, including humidity levels, potential additives present in the polyamide, remnants of molding wax, and other data typically unknown for any given polymer. Additionally, the stability of the added reinforcement also influences this process.

At the initial stages of the process, variable behavior is evident, stabilizing around 20% to 25% decomposition. Upon comparison with the PA11 matrix, it is observed that within the stabilized zone (ranging from 20% to 80% decomposition), all composites exhibit energy values similar to PA11, except for PA11_2CF, which shows a slightly higher value. However, in nearly all cases, less energy is required to initiate the process, increasing until stabilization at 15% of the conversion degree. This trend is consistent except for the composite containing 2CF, where a notable energy requirement (800 kJ/mol) is observed initially, followed by a rapid decrease to levels similar to the others. This phenomenon may be attributed to the enhanced stability of the carbon fiber layers impregnated with the polyamide. Beyond 80% conversion, there is a rise in E_d_ for PA11 and the PA11_CF + EP LPP material, which required less E_d_ in the intermediate zone (258 kJ/mol). Conversely, PA11_2CF and PA11_CF + EP experience a slight decrease. These fluctuations in E_d_ towards the end of the decomposition reaction may be linked to variations in the anchoring between the matrix and the fibers, where improved anchoring results in higher E_d_. However, being an empirical method, significant differences cannot be conclusively stated.

Table 1 illustrates that PA12 exhibits a slightly lower E_d_ compared with PA11 (9 kJ/mol). Figure 3b showcases the E_d_ curves obtained through MFK. With this model, E_d_ for PA12 and PA11 at 50% decomposition are similar (258 and 249 kJ/mol, respectively), but significantly increase for PA12_2CF and PA12_ CF + EP LPP (325 and 287 kJ/mol, respectively). However, a decrease in E_d_ is observed in the case of PA12_CF + EP (182 kJ/mol), a trend not observed in the Kissinger model. CF + EP without LPP treatment presents a lower adhesion with the matrix, which is more evident for PA12, and thus, E_d_ decreases.

Moreover, at the initial stages of the reaction, a substantial amount of energy is required to initiate the process, approximately 1300 kJ/mol for PA12 and 800 kJ/mol for PA12_2CF. The E_d_ at the onset of the reaction is 60 kJ/mol for PA12_CF + EP and 160 kJ/mol for PA12_CF + EP LPP. During this initial phase, the influence of recycled material on decomposition is evident, as it promotes the process. Towards the end of the reaction, no additional energy contribution is necessary for the reaction to conclude in all cases; the required energy decreases slightly upon reaching approximately 90% E_d_. Additionally, although this study shows the behavior of weight as a function of temperature and shows stability up to around 400 °C, it is important to remember that as these materials are heated, they undergo phase transitions and fusion processes. Some of its components could tend to require higher or lower activation energies. These changes in activation energy do not imply weight loss but rather changes in the energy values necessary for the decomposition process, depending on whether they have been doped or have gone through crystallization processes.

The E_d_ curve against the degree of conversion enables the program to conduct isothermal simulations at various temperatures, as outlined in Table 3. In this scenario, only 5% conversion was simulated from Figure 3, aligning with the percentage utilized in the Toop equation (Equation (2)), which suggests that material life ends at 5% decomposition. It is evident that increasing the temperature decreases the time required for complete sample degradation.

Among the materials tested, PA12_2CF exhibits the longest duration to reach 5% decomposition at any given temperature, followed by PA12-CF + EP LPP, PA11_CF + EP LPP, and then PA11. Conversely, PA11_CF + EP requires the shortest time. There is no fixed criterion for the two matrices; however, it appears that composites featuring plasma-treated CF + EP reinforcement necessitate more time for degradation, while untreated CF + EP degrades earlier. This could also be attributed to the fact that compounds treated with plasma increase their melting temperature compared with that of those that have not been treated.

### 3.2. Estimation of the Useful Life of Composite Materials

The estimation of service life for the base polymers PA11 and PA12, along with their respective composites, is directly graphed from the MFK simulation at 5% decomposition and temperature (Table 3). These curves are compared with those calculated using the Toop equation (Equation (2)), with the value of E_d_ at 5% being calculated accordingly. The temperature at 5% decomposition is derived from the degree of decomposition curves (Figure 2c). Table 4 presents the calculated values of E_d_ at 5%, indicating significantly lower values in the composites compared with the pure polyamides, which is particularly evident in the composites featuring C F+ EP, aligning with the observed low adhesion between the matrix and reinforcement.

**Figure 4:**
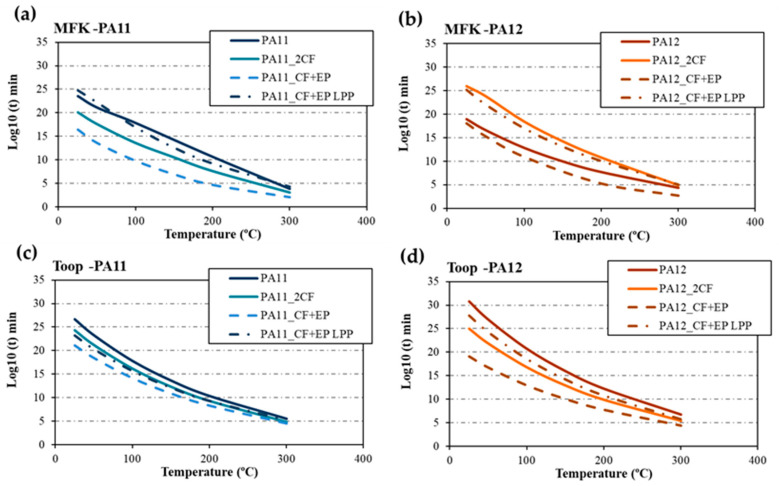
Lifetime estimation by the MFK method for (**a**) PA11 and its composites and (**b**) PA12 and its composites. By Toop’s method for (**c**) PA11 and its composites, and (**d**) PA12 and its composites.

Figure 4 depicts the results of the analysis conducted using both methods. While the values obtained are quite similar, subtle differences emerge because of the empirical nature of the methods employed. The Toop method tends to be more conservative, yielding slightly higher values in certain cases, as observed in the data for PA12 (Figure 4b,d). Nonetheless, the overall trends remain stable irrespective of the method utilized. Specifically, it is evident that the CF+EP material consistently exhibits the lowest curve across all scenarios, indicating shorter degradation times, comparatively. For instance, at 25 °C, PA12_CF + EP would degrade in approximately 2.28 × 10^13^ years, while PA12_CF + EP LPP would require around 1.22 × 10^22^ years for degradation.

**Figure 5:**
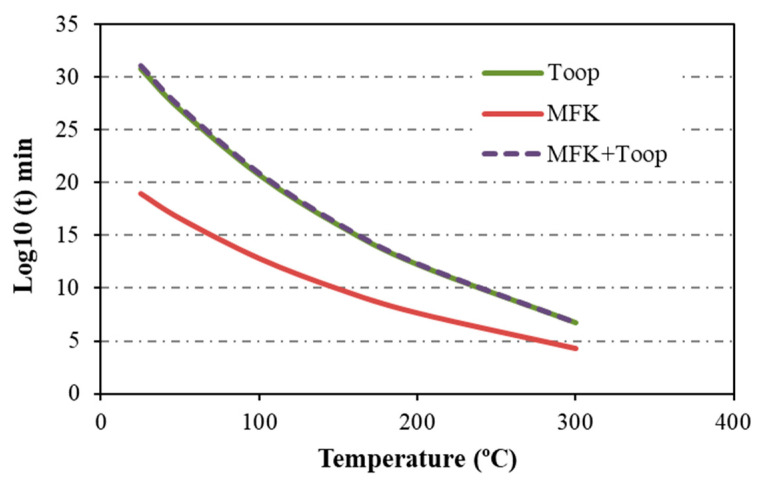
Estimation of the lifetime by the MFK method and subsequent application of Toop for PA12.

In terms of comparison, MFK offers a quicker approach, yet the disparity between methods needs consideration. The Toop method, being more conservative, relies on empirical data that may introduce a similar margin of error. Additionally, the Toop equation was applied to the 5% E_d_ derived from the MFK curves depicted in Figure 3, yielding results consistent with those obtained using Equation (2) and subsequently analyzed by Toop (Figure 5).

**Figure 6:**
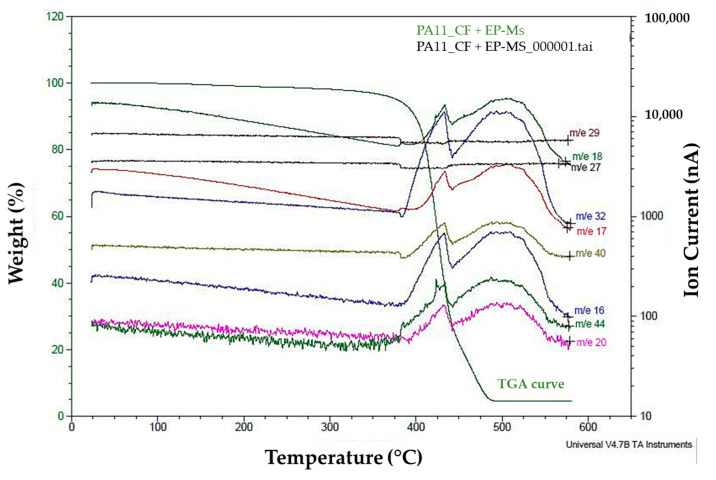
Mass spectroscopy of PA11_CF + EP during the decomposition process.

The mass spectrometry conducted during the temperature elevation offers insights into the gases produced throughout the decomposition process (Figure 6). These released gases correspond to the various substances formed as the material decomposes and are represented as transverse lines or curves running from left to right in Figure 6. The gases that are in the upper part require a greater ionic current to be detected and therefore are the first to be released from the material, and they are also present in greater quantities than those that require a lower ionic current and are located in the upper part of the bottom of the spectrum.

For polyamide 11 and its composite materials, a peak emerges around 370 °C, reaches its peak intensity at 430 °C, and ends around 450 °C. Beyond this point, PA11 exhibits a minor band extending from 450 °C to 500 °C. PA11_2CF and PA11_CF + EP LPP lack this minor band, whereas PA11_CF + EP displays a broader band stretching from 450 °C to 600 °C. The presence of this band after the main peak suggests the continuous release of gases until the conclusion of the test, consistent with the splitting observed in the derivative of the thermogram in Figure 2b.

Although the same gases are released for polyamide 11 and its composites, the major ones are not always the same. If the peak at 430 °C is taken as a reference, for PA11, the majors are H_2_O, O_2_, OH^−^, and CH_4_ (17% each); for PA11_2CF, the majors are H_2_O and OH^−^ (19% each), with O_2_ and CH_4_ released at 14% and 15%. In the case of PA11_CF + EP, the major is O_2_ (25%), CH_4_ (18%), CO_2_ (15%), hydroxyls (11%), and H_2_O (8%). With plasma treatment on the recycled material, the predominantly released gas is CO_2_ (34%), followed by H_2_O (15%), O_2_, OH^−^, and CH_4_ (13% each). Minor gases such as the “oxydanodium cluster” and the polymer chain of three carbons are also released.

Moreover, for polyamide PA12 in mass spectrometry, a singular peak emerges, commencing at 300 °C, reaching its maximum at 450 °C, and ending around 500 °C. Occasionally, a shoulder within the primary group appears at 350 °C. Despite not producing two distinct peaks in the spectrum derivative, the primary gases remain O_2_ (24%), H_2_O (18%), CH_4_ (18%), OH^−^ (17%), and CO_2_ (14%) (Table 5).

The emission gas spectra peaks for PA12_2CF are 15° higher than those for PA12 because of the presence of carbon fibers, which enhance material stability (Table 5). The shoulder observed in some PA12 spectra becomes more pronounced, particularly for O_2_ and CH_4_. The approximate gas proportions are O_2_ (25%), CO_2_ (21%), CH_4_ (20%), H_2_O (15%), and OH^−^ (11%).

When the reinforcement consists of recycled material PA12_CF+EP, only one peak is observed. CO_2_ starts releasing earlier at 350 °C, with the other gases released around 425 °C, peaking at 485 °C, and concluding around 510 °C. CO_2_ is the primary gas (45%), followed by O_2_ (20%), CH_4_ (18%), OH^−^ (9%), and H_2_O (8%). Minor gases like H_4_O_2_^+^ and -CH-CH-CH_2_- are absent in this composite. With plasma-treated recycled material PA12_CF + EP LPP, the released gases and their positions remain unchanged, with a 35° delay compared with PA11. The proportions are similar to those of PA11_CF + EP as follows: CO_2_ (42%), O_2_ (23%), CH_4_ (17%), OH^−^ (9%), and H_2_O (9%).

The 5% degradation of materials occurs above 350 °C for PA11 and above 400 °C for PA12. However, gas release initiates from 300 °C, containing CO_2_ and CH_4_ from polyamides, although in minimal quantities, as the observed peak reaches higher temperatures (Table 5). This validates the selection of 5% decomposition to apply the Toop equation and estimate the lifespan of these materials.

## 4. Conclusions

This study investigated the thermal degradation behavior of mechanically recycled carbon fiber composites (rCF) containing intact epoxy resin, using PA11 and PA12 as thermoplastic matrices. Untreated polyamides and virgin carbon fiber composites served as references.

Thermogravimetric analysis (TGA) with MFK and Kissinger models highlights significant differences in degradation temperatures (T_d_) and decomposition energies (E_d_) between PA11 and PA12. These variations likely stem from dopant compounds added during manufacturing processes, affecting crystallization, fusion, and ultimately, lifespan.

PA11 consistently exhibited lower T_d_ values, indicating faster initial degradation compared with PA12. However, Kissinger-calculated E_d_ values were higher for PA11, revealing different decomposition pathways based on the reinforcement type.

Further insights emerged from the MFK model, which depicted variations in E_d_ at 50% decomposition, providing insights into energy requirements for different stages of the degradation process. The estimated lifespans, based on E_d_ at 5% decomposition, showed that rCF materials had shorter lifespans because of inferior adhesion to the polyamide matrix. The Kissinger + Toop method provided more conservative estimates, projecting longer times.

Additionally, analysis of decomposition gases identified water, oxygen, carbon dioxide, hydroxyls, methane, and smaller gases. The proportions of these gases varied with the reinforcement type, suggesting intricate relationships between composition and decomposition products. In all cases, they were released from 300 °C, with proportions dependent on the composite’s reinforcement. In summary, the use of rCF as reinforcement can preserve and even improve the thermal stability of composites, especially if there is a strong adhesion between the rCF and the matrix. Additionally, combining rCF with bio-based thermoplastic matrices like PA11 offers an environmentally friendly approach to composite development.

## Figures and Tables

**Table 1:** T_d_ for PA11 and PA12 polymers and their composites.

Polyamides/Composites		PA	2CF	CF + EP	CF + EP LPP
**T_d_ (°C)**	PA11	426 ± 8	431 ± 8	429 ± 8	426 ± 9
	PA12	454 ± 10	462 ± 8	465 ± 7	463 ± 11

**Table 2:** E_d_ values according to the Kissinger model for PA11 and PA12.

Polyamides/Composites		PA	2CF	CF+EP	CF+EP LPP
**E_d_ (kJ/mol)**	PA11	290.32	288.50	248.16	283.11
	PA12	241.89	237.66	261.53	244.26

**Table 3:** Isothermal simulation at 5% decomposition from the MFK.

Applied Kinetics: Conversion								
Material	α (%)	Time	Temperature (°C)					
			25	50	100	150	200	300
PA11	5	min	3.48 × 10^23^	1.30 × 10^21^	6.95 × 10^17^	1.85 × 10^15^	4.47 × 10^10^	7255
PA11_2CF			1.17 × 10^20^	3.67 × 10^17^	3.64 × 10^13^	3.18 × 10^10^	3.82 × 10^7^	1090
PA11_CF + EP			2.70 × 10^16^	3.98 × 10^13^	6.54 × 10^9^	8.81 × 10^6^	5.17 × 10^4^	127
PA11_CF + EP LPP			6.18 × 10^24^	1.63 × 10^22^	8.62 × 10^16^	4.04 × 10^12^	1.79 × 10^9^	21,180
PA12			8.95 × 10^18^	3.90 × 10^16^	6.56 × 10^12^	8.61 × 10^9^	4.59 × 10^7^	20,240
PA12_2CF			9.54 × 10^25^	6.71 × 10^23^	2.65 × 10^18^	1.69 × 10^14^	5.95 × 10^10^	79,100
PA12_CF + EP			1.05 × 10^18^	1.78 × 10^15^	7.79 × 10^10^	1.67 × 10^5^	4.60 × 10^2^	21,200
PA12_CF + EP LPP			1.49 × 10^25^	1.08 × 10^22^	9.71 × 10^16^	1.36 × 10^13^	1.24 × 10^10^	119,000

**Table 4:** E_d_ values at 5% according to the Kissinger model for PA11 and PA12.

Polyamides/Composites		PA	2CF	CF + EP	CF + EP LPP
**E_d_ (kJ/mol)**	PA11	261.08	231.94	196.73	214.97
	PA12	303.85	232.96	174.29	262.46

**Table 5:** Temperatures and allocation of gases released during the test.

PM (g/mol)	Polyamide	16	17	18	20	32	40	44
Gas		CH_4_	OH^−^	H_2_O	H_4_O^2+^	O_2_	−CH−CH−CH_2_	CO_2_
	PA11	430	430	430	430	432	430	430
Temperature (°C)	PA11_2CF	430	430	430	430	432	430	430
	PA11_CF + EP	430 500 *	430 500 *	430 500 *	430 500 *	432 500 *	430 500*	430 500 *
	PA11_CF + EP LPP	430	430	430	430	430	430	430
	PA12	350 450	450	450	450	350 450	450	350 450
	PA12_2CF	365 465	465	465	465	365 465	465	465
	PA12_CF + EP	485	485	485	—	485	—	485
	PA12_CF + EP LPP	485	485	485	—	485	—	485

500 * banda ancha.

## Data Availability

The raw data supporting the conclusions of this article will be made available by the authors on request.
